# Seroprevalence of Hepatitis B Virus and Associated Factors among Health Professionals in University of Gondar Hospital, Northwest Ethiopia

**DOI:** 10.1155/2019/7136763

**Published:** 2019-03-03

**Authors:** Aynishet Adane Gebremariam, Adino Tesfahun Tsegaye, Yalelet Fentaw Shiferaw, Mebratu Mitiku Reta, Alem Getaneh

**Affiliations:** ^1^Department of Internal Medicine, School of Medicine, College of Medicine and Health Sciences, University of Gondar, Gondar, Ethiopia; ^2^Department of Epidemiology and Biostatistics, Institute of Public Health, College of Medicine and Health Sciences, University of Gondar, Gondar, Ethiopia; ^3^University of Gondar Comprehensive Specialized Hospital, Gondar, Ethiopia; ^4^Department of Medical Microbiology, School of Biomedical and Laboratory Sciences, College of Medicine and Health Sciences, University of Gondar, Gondar, Ethiopia

## Abstract

**Introduction:**

Hepatitis B virus infection is one of the commonest occupational risks in healthcare workers. However; there is limited evidence regarding the prevalence of hepatitis in health professionals in Ethiopia.

**Objective:**

This study was aimed at assessing the prevalence of hepatitis B and associated factors in health professionals.

**Methods:**

Institution based cross-sectional study was conducted among health professionals at University of Gondar Hospital from January to February, 2015. Self-administered questionnaire was used to collect sociodemographic variables and blood sample was also taken to determine hepatitis B virus sero-status. Chi square test with 95% confidence interval (CI) was computed to assess the associations of different factors with hepatitis B infection.

**Result:**

A total of 332 health professionals (with a response rate of 92.2%) participated in the study. Most (98.5%) of health professionals were not vaccinated for hepatitis B. The prevalence of hepatitis B in health professionals at UOG hospital was found to be 4.52% (95% CI: 2.4, 6.5). Hepatitis B infection was more common among males (P value =**0.0299).** * Conclusion*. The prevalence of hepatitis B in health professionals in this study was comparable with other studies done in Ethiopia among health professionals. Males were more affected than females for hepatitis B infection. Hepatitis B virus vaccine, treatment for the infected, and training on infection prevention should be more available for healthcare workers.

## 1. Introduction

Hepatitis B virus (HBV) is a small double-stranded DNA virus which predominantly affects the liver. It is a global public health problem accounting for more than 300 million HBV carriers worldwide and is found to be high in the developing countries particularly in Asia and Sub-Saharan Africa with a prevalence rate of 10-20%. HBV is one of the most common causes of acute and chronic liver disease [1].

Different community-based studies conducted in Ethiopia showed that the prevalence of hepatitis B seropositivity (HBsAg) ranges from 5 to 11% [2–7]. Although the modes of transmission of HBV vary in different geographical areas, the main routes of transmission are sexual intercourse, parenteral contact, and vertical transmission [8]. A study done in Ethiopia showed that the predominant modes of HBV transmission were found to be horizontal intrafamilial spread, harm full traditional practices, and use of unsterile materials [9].

HBV is one of the most commonly transmitted blood-borne viral infections in healthcare settings; hence it is the leading issue of concern particularly in resource-limited healthcare settings. However, with routine vaccination of healthcare workers (HCWs) for HBV and routine use of standard infection prevention measures, the incidence of HBV infection is decreasing in HCWs [10, 11]. Diagnosis of hepatitis B can be made primarily by the detection of HBsAg, anti- HBcAb, anti-HBs antibodies, and HBV DNA levels in the serum [12]. Although there are few studies done in Ethiopia on the prevalence of hepatitis B at a community level, there is a paucity of evidence regarding the prevalence of hepatitis B infection in healthcare workers, particularly in the study area. Therefore, this study was intended to assess the seroprevalence of HBV infection and associated factors in health professionals working at the University of Gondar Hospital (UOGH), Northwest Ethiopia.

## 2. Methods and Materials

### 2.1. Study Design and Setting

An institution-based cross-sectional study was conducted from January to February 2015. The study was done at the University of Gondar Hospital, which is located 748 kilometers in the Northwest of Addis Ababa, the capital of Ethiopia. The university hospital is both a teaching and referral hospital serving about 5 million people in its catchment area. In the setting, there is no routine vaccination of health professionals for HBV.

### 2.2. Participants

All health professionals working at the University of Gondar were included with a total sample size of 332. Structured and pretested self-administered questionnaire was used to collect information regarding sociodemographic variables and possible risk factors for hepatitis B infection acquisition. After informed written consent was obtained from study subjects, about 3 milliliter (ml) of venous blood sample was collected aseptically from each of the study participants. Serum was separated from each sample after centrifugation and stored in the refrigerator at -20°c for a maximum of 03 days until analyzed for HBsAg and samples were labeled with unique identification numbers which are similar to the code given in the questionnaire. The serum was analyzed for HBsAg using rapid test kits (Bio-Dignos Biotechnology co., Ltd.P.R.China) following the manufacturer protocols. Samples reported to be positive for HBsAg by the rapid kits were double checked using ELISA (Anthos reader, 2001) test for final confirmation at the main laboratory of University of Gondar Hospital. Samples which became positive by the ELISA test were reported as positives.

### 2.3. Data Analysis

Data were entered to EPI-INFO software version 3.5.3 and analyzed by using SPSS software version 20. Descriptive statistics were used to see the prevalence of HBV serostatus and other associated variables. Chi-square test was used to see the association of independent variables with the dependent variable.

### 2.4. Ethics Approval

Ethical clearance was obtained from the ethical review board of the University of Gondar. Written permission letter was obtained from the University of Gondar Hospital. After explaining the aim of the study, informed written consent was taken from each participant. Confidentiality was maintained at all levels of the study by avoiding any personal identification. After doing serum analysis, counseling was given for participants who were infected with hepatitis B virus, and they were referred for further workup and treatment for hepatitis B. Participants who were negative for HBV were vaccinated for hepatitis B.

## 3. Result

### 3.1. Sociodemographic Characteristics of Respondents

A total of 332 health professionals have participated in the study. The median age of participants was 27 years (Interquartile Range (IQR), 4). Hundred and ninety-eight (59.6%) were males and 226 (68.07%) of the participants were unmarried. Regarding the occupation of study participants, 186 (56.0%) were clinical nurses followed by physicians accounting 60 (18.1%) observations. The median duration of work experience of participants was 3 years (IQR, 3.84). As far as the place of work is concerned, 106 (31.9%) of participants were practicing in the department of surgery and 66 (19.8%) in internal medicine (Table 2).

### 3.2. Exposure to Potential Risk Factors for HBV Infection

Out of 332 participants, only 11 (3.3%) were partially vaccinated for HBV and only 5 (1.5%) of the participants had a history of transfusion. History of parenteral injection was reported by 161 (48.5%) of the participants, whereas about 144 (43.4%) of the participants had intimate contact with jaundiced persons. Majority of participants (194 (58.4%)) reported a previous history of needle prick injury and 269 (81%) had blood contact with their skin or mucosa during clinical practice. Fifty-five (16.6%) of participants reported the previous history of traditional harmful practices including scarification, tattooing, ear piercing, traditional circumcision, and traditional tonsillectomy. From all of the participants, 11 (3.3%) of them reported the previous history of having multiple sexual partners (Table 1).

### 3.3. Prevalence of Hepatitis B Infection in Health Professionals

According to this study from 332 HCWs who participated, the prevalence of hepatitis B infection was found to be 15/332: 4.52% (95%CI: 2.4, 6.5). The prevalence has variability across different factors. It is 5.15% (95% CI: 2.7, 8.8) among HCWs with work experience of > 2 years and 3.03% (95% CI: 0.63, 8.60) in those with work experience of <= 2 years. Among the infected health professionals 86.7% were males and 66.7% were found in the age group between 26 and 30 years (Table 2). The prevalence of HBV infection in males was 6.57% and it has statistically significant difference with females 1.49% (P<0.0299).

### 3.4. Prevalence of HBV among HCWs by Behavioral Factors

**Figure 1:**
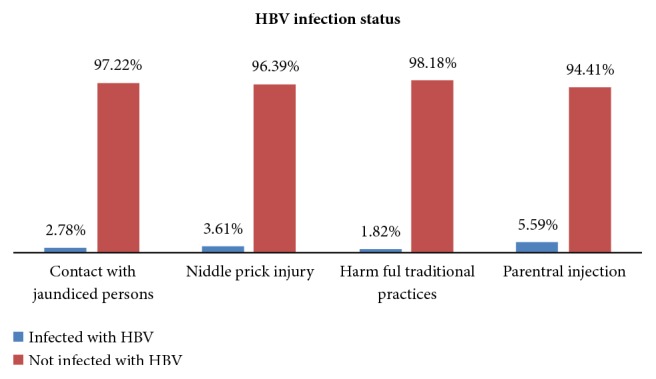
Prevalence of HBV infection with respect to different behavioral factors among HCWs at University of Gondar Hospital, Northwest Ethiopia, 2015.

Among 269 HCWs who had history of blood contact with their skin and/or mucosa, 12 (4.5%) were infected by HBV. There were also 5 HCWs who had history of blood transfusion and 2 of them (40%) had HBV infection. Eleven HCWs were vaccinated for HBV partially and among them 1 (9.1%) had HBV infection. The prevalence was also 2.78% in HCWs who had intimate contact with jaundiced persons (Figure 1).

## 4. Discussion

In Ethiopia, there are only limited studies done on the seroprevalence of hepatitis B among health professionals. Hence, this study was conducted with an intention to assess the magnitude of hepatitis B and its risk factors. According to this study, the seroprevalence of hepatitis B based on HBsAg seropositivity was 4.52% (95% CI: 2.4, 6.5).

The prevalence of hepatitis B in this study is consistent with studies done in Korea (2.4%), Turkey (3.0%), and Pakistan (5.0%) and a study done in Sudan (6.0%) [13–16]. It is also consistent with studies done among health professionals in Northeast, Northwest, and Central Ethiopia (with a range of 2.4 to 7.3%) [10, 17–20]. The prevalence in this present study is higher than the studies done in Germany (2.2%), Brazil (0.8%), Mexico (1.2), and Yemen (1.5%) [21–24]. This could be explained by variation in the study settings. The other studies were conducted in more developed countries where healthcare professionals get appropriate vaccines and work in a better clinical setting where their safety is relatively maintained. On the other hand, the prevalence was lower than the other studies done in Senegal (17.8%), Uganda (8.1%), and Nigeria (11.3%) [25–27]. It could be due to differences in the epidemiology of hepatitis B infection in the respective study areas, baseline sociodemographic characteristics of the study subjects, or difference in vaccination status or variation in the timing of the study period. If we compare with studies done in Ethiopia, the prevalence of hepatitis B in health professionals in this study is relatively lower than what is reported from studies done in Bule Hora Wereda (7.3%) and Addis Ababa (9.0%) [10, 28]. The settings of these studies have no significant difference from the current study. However, differences could happen due to diagnostic techniques and some methodological variations. In addition, the variation could be explained by the approaches used by the studies.

According to this study, only 3.3% of study participants were partially vaccinated for hepatitis B. This means that they took less than the recommended three doses. Concerning the other factors, 48.5% of participants had a history of parenteral injection; 43.4% of participants had intimate contact with jaundiced persons during clinical practice; 58.4% had needle prick injury; and 16.6% participants had harmful traditional practices; however, none of these factors were associated with hepatitis B infection status.

Males were more affected than females for hepatitis B infection (P value:** 0.0299)**. This finding was in line with studies done in Turkey [25], Bule Hora Wereda [10], and Addis Ababa [28]. This could be explained by variations to different exposure factors. Males are more likely to have outdoor exposure to factors that could increase their risk of hepatitis B infection.

## 5. Conclusion

In conclusion, the prevalence of hepatitis B in health professionals in this study was comparable with other studies done in Ethiopia among health professionals and most of the health professionals who participated in this study were not vaccinated for hepatitis B. Males were more affected than females for hepatitis B infection. Hepatitis B virus vaccination, treatment for the infected, and training on infection prevention should be more available for healthcare workers

## Figures and Tables

**Table 1:** Exposure to potential risk factors or HBV infection among HCWS at UOG hospital, Northwest Ethiopia, 2015.

**Variables **	**Number**	**Percent**
**Vaccination status **		
Yes	11	3.3
No	321	96.7
**Blood transfusion **		
Yes	5	1.5
No	327	98.5
**Parenteral injection **		
Yes	161	48.5
No	171	51.5
**Contact with jaundice person **		
Yes	144	43.4
No	188	56.6
**Needle prick injury **		
Yes	194	58.4
No	138	41.6
**Blood contact with skin or mucosa **		
Yes	269	81
No	63	19
**Harmful practice **		
Yes	55	16.6
No	277	83.4
**Unprotected sex**		
Yes	33	9.9
No	299	90.1
**Multiple sexual partnerships**		
Yes	11	3.3
No	321	96.7

**Table 2:** The prevalence of HBV among health professionals by sociodemographic variables at University of Gondar Hospital, Northwest Ethiopia, 2015.

**Variables **	**HBV serostatus**		**Total**	**Chi square test (corrected)**
	**Yes**	**No**		**P-value**
	**Frequency (**%**)**	**Frequency (**%**)**	**Frequency (**%**)**	
**Sex **				

Male	13(6.57)	185(93.43)	198 (59.6)	**0.0299**

Female	2(1.49)	132(98.51)	134 (40.4)	

**Age (Years)**				

<=25	3(2.8)	104(97.2)	107 (32.2)	0.5688

26-30	10(5.5)	172(94.5)	182 (54.8)	

>30	2(4.65)	41(95.35)	43 (13.0)	

**Marital status**				

unmarried	12(5.3)	214(94.7)	226 (68.07)	0.312

Married	3(2.8)	103(97.2)	106 (31.93)	

**Profession**				

Physician	3(5.0)	57(95.0)	60 (18.1)	0.3499

Clinical nurse	8(4.3)	178(95.7)	186 (56.0)	

Midwife	0 (0.0)	27(100.0)	27 (8.1)	

Laboratory Technician	2(4.44)	43(95.56)	45 (13.6)	

Anesthetist	2(14.3)	12(85.7)	14 (4.2)	

**Work experience **				

< = 2 years	3(3.03)	96(96.97)	99 (29.8)	0.3964

>2 years	12(5.15)	221(94.85)	233 (70.2)	

**Department **				

Internal medicine	3(4.55)	63(95.45)	66 (19.88)	0.624

Surgery	8(6.06)	124(93.94)	132 (39.76)	

Obstetrics and Gynecology	0(0.0)	39(100.0)	39 (11.7)	

Pediatrics	2(4.0)	48(96.0)	50 (15.1)	

Laboratory	2(4.44)	43(95.56)	45 (13.6)	

## Data Availability

The data used to support the findings of this study are available from the corresponding author upon request.
